# Creative webs: decoding the creativity potential of Twitter followers

**DOI:** 10.3389/fpsyg.2024.1294838

**Published:** 2024-05-01

**Authors:** Tsahi Hayat

**Affiliations:** Sammy Ofer School of Communications, Reichman University, Herzliya, Israel

**Keywords:** creativity, social network analysis, Esports, trends, Twitter

## Abstract

**Introduction:**

Previous studies have associated creativity to one’s social networks. This study builds on this framework and examines the premise that one’s social fabric is a catalyst for creativity, specifically probing the dynamics between online social connections and creative expressions in the realm of Esports. Therefore, this research paper examined a correlation between people’s creativity and their Effective Network Size (non-redundant ties) on Twitter, to see if potentially non-redundant information is related to creativity.

**Methods:**

Creativity score was defined as the propensity of a participant to utilize specific terms relating to Esports in its emerging stages, prior to its peak popularity as evidenced by Google Trends. Effective Network Size was analyzed based on the social ties of participants on Twitter (*N* = 50,000).

**Results:**

The findings indicate that the higher the Effective Network Size score, the higher the creativity score. Furthermore, geographically dispersed social networks moderated the relationship between Effective Network Size and creativity. For people with more dispersed social networks, ENS was more constructive for creativity.

**Discussion:**

These findings are discussed in the broader context of the relevancy of online social networking sites for creativity.

## 1 Introduction

Creativity is a pivotal asset, serving both individuals in tasks like problem-solving and society at large by driving scientific discoveries, artistic movements, inventions, and economic advancements (Sternberg and Lubart, 1999). Historically, creativity was often explored in relation to other personal traits (Barron and Harrington, 1981). Yet, over time, the focus expanded to encompass the role of social factors in fostering creativity. Research suggests that social interactions significantly shape the creative process (Perry-Smith and Shalley, 2003). Burt (2004) posited that individuals with diverse social networks are more likely to access unique information, thereby transforming creativity into a dynamic exchange of ideas.

While prior studies have delved into the link between face-to-face social networks and creativity (Perry-Smith, 2006; Zhou et al., 2009) or the relationship between online social networks and social capital (Hofer and Aubert, 2013; Hayat et al., 2017a), our research pioneers the exploration of the association between online social networks, specifically Twitter, and creativity. Twitter, a platform where users can freely connect, offers an environment conducive to expanding non-redundant social ties, potentially exposing users to a diverse array of information and, in turn, bolstering creativity (Kwak et al., 2010). Utilizing a custom script, we gathered data on participants’ Twitter connections, calculating a non-redundant ties score for each (i.e., a user’s number of friends that are not connected among themselves). We then assessed the participants’ creativity by looking at how early they mentioned trending ideas in their tweets (compared to when these ideas peaked). Our findings indicate a positive correlation between non-redundant ties and creativity scores.

This research underscores the significance of online social networking sites, not just in the realm of social capital but also in the domain of creativity. By leveraging Twitter, we have illuminated social mechanisms previously not examined in traditional studies.

## 2 Related work

### 2.1 Creativity

In a foundational work, Amabile (1983, p. 359–360) defines creativity as:

“A product or response is deemed creative when independent observers concur on its creativity. Such a product or response is judged creative if (a) it presents a novel yet appropriate, useful, correct, or valuable solution to the given task, and (b) the task is heuristic in nature rather than algorithmic.”

Creativity, can further be defined as the capacity to generate or recognize ideas, alternatives, or possibilities that may be useful in solving problems, communicating with others, and entertaining ourselves and others. Furthermore, recognizing the value of new information, assimilating it, and communicating it requires a blend of creativity and adaptability (Cohen and Levinthal, 1990). The Absorptive Capacity Theory, proposed by Cohen and Levinthal (1990), posits that individual’s ability to recognize the value of new information, assimilate it, and apply it to commercial or creative ends is pivotal for innovation. Specifically, the act of identifying and discussing trending topics requires a similar process. The recognition of a trending topic among a vast array of information demands a keen sense of what is relevant and potentially valuable. This discernment is akin to the first step in the absorptive capacity – the ability to recognize and value new information. Secondly, assimilating this information involves understanding the context and nuances of the trend, which is critical for meaningful engagement. Lastly, creatively applying or discussing these trends can be seen as the third step, where individuals adapt this information in unique ways, be it through insightful comments, creative hashtags, or linking trends to other concepts. This process demonstrates creativity, as it involves not just the consumption of information but its novel application in a dynamic social media environment. Hence, following previous work, we will look at the individuals references to trending topics as a mean to gain insight into creativity (Ceh et al., 2023).

Historically, the research spotlight was on the personality traits (or human capital) linked with creativity (Barron and Harrington, 1981). Over time, the lens broadened to encompass social factors that bolster creativity, emphasizing the role of social interactions in shaping the creative process (Perry-Smith and Shalley, 2003). The benefits accrued from being part of a social network are encapsulated in the term ‘social capital’ (Bourdieu, 1986). Social capital offers insights into how social ties can enhance creativity by introducing individuals to a diverse range of perspectives (Perry-Smith, 2006). It also underscores the potency of structural holes in delivering non-redundant information (Burt, 1992). A structural hole is characterized by a scenario where an individual (ego) is directly linked to two other individuals (alters) who aren’t connected to each other. Such a configuration ensures that the ego receives unique information (Burt, 1992). This concept is pivotal to our study as we delve into the nexus between individuals’ online social networks, their creativity, and the social capital emanating from their varied online connections.

### 2.2 Social networks

A social network is characterized as a collection of individuals (or organizations or other social entities) linked by various social relationships, such as friendship, collaboration, or information exchange (Garton et al., 1997). Expanding on this, it can be described as a framework comprising actors (nodes) and their interrelationships (links) within a societal context (Wasserman and Faust, 1994, as cited in Chung et al., 2016). Two primary analytical approaches exist: whole network analysis, which examines ties across all network members (Wellman, 2007), and ego network analysis. The latter delves into relationships from the vantage point of a specific node, termed the ‘ego’—in our context, this refers to a particular Twitter user perspective.

Social network analysis emphasizes structural evaluation, where the primary unit of study is the relationship itself (Garton et al., 1997). This method seeks to map and quantify various relationships—be they formal, informal, weak, or strong—to scrutinize information flows (Serrat, 2017) or resource exchanges, and to discern the distinct impacts such networks exert on individuals or entities (Garton et al., 1997). Resources garnered from these networks, often termed social capital, encompass valuable information, personal relationships, and the capacity to mobilize groups (Paxton, 1999). To truly grasp the interplay between social networks and creativity, it’s imperative to understand the relationship between social networks and social capital.

### 2.3 Social capital

Social capital represents the anticipated benefits derived from social relationships (Coleman, 1988). Essentially, it encapsulates the advantages one gains from being part of a social network (Bourdieu, 1986). Illustratively, Coleman (1988) highlighted how the tight-knit relationships among Jewish diamond merchants in New York, fortified by practices like intermarriage, fostered a trust so profound that diamonds could be exchanged without formalities or fears of theft.

Wellman (2001), suggest that social capital can also be cultivated through online interactions. For instance, Hayat et al. (2017b) revealed that online social ties can offset limited eHealth literacy. By connecting with users facing similar health challenges, participants, especially those from ethnic minorities, reported enhanced perceived health outcomes.

Broadly, social capital bifurcates into bonding and bridging capital. Bonding capital emerges from strong, intimate connections within socially cohesive groups, like families or close friends. In contrast, bridging capital arises from weaker ties within socially diverse groups, such as colleagues or business associates (Putnam, 2000; Adler and Kwon, 2002; Ferlander, 2007). This research differentiates between bridging capital, formed from a mix of weak and diverse ties, and mere weak ties. This distinction is pivotal as our focus is on tie diversity rather than strength.

Bridging capital’s strength lies in its potential to introduce new information via diverse contacts (Putnam, 2000). For instance, job seekers often benefit more from weak social connections, which can unveil opportunities beyond their immediate circles (Granovetter, 1977). Conversely, bonding capital offers emotional sustenance, like the support one receives from close friends during challenging times.

Perry-Smith and Shalley (2003) posited that weak ties, by introducing diverse information and viewpoints, can enhance creativity. While not all information from weak ties is inherently creative, it’s often novel and less redundant, thus potentially sparking creativity (Perry-Smith, 2006). Zhou et al. (2009) identified a nuanced relationship between weak ties and creativity, noting optimal creativity when employees maintained a moderate number of weak ties. Interestingly, they did not observe a direct negative correlation between strong ties and creativity. They hypothesized that while strong ties offer trust and support, fostering creativity, they might also limit diverse perspectives due to homophily (Obstfeld, 2005), potentially constraining creative thought.

Perry-Smith (2006) further emphasized the role of weak ties in fostering creativity among scientists, though this relationship was mediated by background diversity. This finding was replicating more recently (Hayat et al., 2020, 2021). In summary, weak ties emerge as a significant catalyst in nurturing creativity.

### 2.4 Online bridging social capital on Twitter diversified social networks

Kwak et al. (2010) note that Twitter’s structure allows users to freely follow profiles without the obligation of reciprocation. This dynamic facilitates rapid expansion of users’ social networks, potentially augmenting their social capital (Hofer and Aubert, 2013). Such a unique friendship model positions this study to probe into the non-redundant relationships of Twitter users.

Hofer and Aubert (2013) observed a correlation between the number of profiles a user follows on Twitter and their online bridging social capital. Essentially, a broader following spectrum offers users a richer tapestry of opinions on their feeds. More recent research ranks Twitter users as having the highest bridging social capital, followed by those on Instagram, Facebook, and Snapchat (Phua et al., 2017). This ranking aligns with Twitter’s inherent design, which encourages users to connect with individuals beyond their immediate real-life circles. The platform’s diverse weak ties can catalyze the dissemination of novel information (Jin and Phua, 2014) and foster interactions with a broader audience (Williams, 2006).

### 2.5 Diversified social networks

While it’s acknowledged that not all weak ties bridge distinct social spheres and not all strong ties are intrinsically interconnected (Zhou et al., 2009), Burt (1992) offered a counterpoint to the ‘strength of weak ties’ paradigm. He introduced a measure of structural holes, termed Effective Network Size, which quantifies non-redundant ties a central node maintains, irrespective of tie strength.

Studies underscore that information predominantly circulates within groups rather than between them. In this context, structural holes emerge as a competitive edge for those whose connections traverse these gaps. These holes present opportunities to mediate information flow between groups, each holding valuable insights for the other (Burt, 2017).

Individuals whose networks are replete with structural holes often find themselves better informed, more involved, and in control of diverse opportunities. Their connections across disparate groups ensure a stream of information with minimal redundancy (Burt, 2017).

In the world of entrepreneurship, entrepreneurs bridge the structural holes in their networks via brokers to achieve bridging capital for the success of their projects (Burt, 2005). A manager who spans a structural hole by having relations with contacts on both sides of a hole can obtain crucial information for his/her organization (Burt, 1997) and is more likely to develop good ideas (Burt, 2004).

Burt’s (2004) work underscores the value of bridging disparate groups, positing that such connections catalyze the dissemination of innovative ideas, effectively transforming creativity into a cross-pollination enterprise. Additionally, those who act as conduits across ‘structural holes’ in social networks often reap professional rewards, including enhanced reviews, accelerated career progression, and increased income (Burt, 2000).

Echoing Burt, Anderson’s (2008) research corroborates the positive impact of expansive social networks on managers’ access to a rich variety of information, as quantified by Effective Network Size. However, Anderson’s findings contest the presumed advantage of weak ties for information diversity. This contradicts earlier assumptions and calls into question the singular effectiveness of weak ties, a challenge also reflected in the work of Hansen et al. (2005).

While some scholars have questioned Burt’s theories, Perry-Smith and Mannucci (2017) among them, the relationship between structural holes and creativity remains contested. Zhou et al. (2009) found no link between these structural holes and creative output, suggesting that the presence of weak ties and structural holes does not guarantee a diverse information set for the individual. This becomes more complex considering that information from a common network might be redundant, regardless of whether the network contacts are themselves connected.

This complexity is compounded by cultural differences. Xiao and Tsui (2007) observed that in China’s collectivist society, the benefits associated with structural holes in Western individualistic cultures do not hold. In these societies, tight-knit networks fostering trust and reciprocity are more valued. Perry-Smith (2006), despite researching an American demographic, also refuted a direct connection between structural holes and creativity, suggesting instead that the advantage lies in the weak ties themselves rather than the information diversity they might offer.

Contrastingly, Anderson (2008) maintains that the value of weak ties lies in their role as bridges across structural holes, following Burt (1992). Yet, when the bridging function is controlled for, Anderson posits that strong ties actually prove more fruitful for information gathering, citing Krackhardt (1992) on their greater tendency to share knowledge willingly.

### 2.6 Diversified social networks online

The impact of diverse social networks is also demonstrated in the online sphere. A recent study has highlighted the importance of one’s Effective Network Size in regards to one’s content popularity on online social networks, especially when the content producer is a woman (Lesser et al., 2017).

Furthermore, the person who shares a piece of information within online social networking sites, can influence the perceived credibility of that content (Samuel-Azran and Hayat, 2019). On an aggregated level, high diversity among people who share a given piece of information on Twitter affects recipients’ credibility assessment regarding that piece of information such that it is perceived as more credible (Hayat and Hershkovitz, 2018; Hershkovitz and Hayat, 2020), especially among recipients with high digital literacy or high *need for cognition* (Hayat et al., 2019) – a personality trait with high cognitive motivation to gather and process information (Anderson, 2008). Such results are alarming due to the prominent rise of social networking sites as news sources. If information’s visual cues are perceived as more important than the information itself, then, now more than ever, ‘the medium is the message’.

To conclude, social parameters play a crucial role in facilitating creativity. However, to our knowledge, no researcher has yet examined a relationship between Twitter users’ creativity and their Twitter social networks such that the more diversified their Twitter social networks the more creative they are. Therefore, the second hypothesis is:

*H1*: Effective Network Size of Twitter users and their creativity are positively correlated.

### 2.7 Remote social connections, information diversity, and creativity

In his influential analysis, Granovetter (1977) introduced the concept that relationships with acquaintances, or “weak ties,” are crucial for accessing a wider array of unique information. These peripheral connections often act as conduits to different social spheres, enhancing the diversity of information one receives. Within the digital era, these weak ties extend beyond physical proximity, enabling the exchange of new ideas across vast geographical divides through online platforms.

As discussed above, Burt (2004) emphasized the concept of “structural holes,” where individuals who bridge gaps between different social groups have a competitive advantage in accessing diverse information. This diversity of information is crucial for creativity as it provides a broader perspective and a richer set of resources for idea generation (Perry-Smith, 2006). Remote connections, by their nature, often span across different cultures, industries, and backgrounds, making them a potent source of diverse information.

The rise of digital platforms like LinkedIn, Twitter, and various academic forums has made it easier to establish and maintain remote connections. These platforms not only facilitate information exchange but also foster collaborative projects that span across borders (Aral et al., 2012). Such collaborations often lead to innovative outcomes due to the amalgamation of diverse perspectives. Hence, our second research hypothesis is:

*H2*: When the geographical distance between an ego and its alters is higher, Effective Network Size of Twitter users and their creativity are more strongly correlated compared to cases where the geographical distance is lower.

The context in which the research hypothesis will be examined, is the evolving field of Esports. The realm of Esports has garnered substantial attention in both academic and popular discourse, reflecting its meteoric rise and cultural significance. Esports, or competitive video gaming, has evolved from a niche pastime to a global phenomenon, engaging hundreds of millions of enthusiasts worldwide. This remarkable growth is not merely a trend within the gaming industry but a pivotal shift in entertainment, social interaction, and economic models. Moreover, the study of Esports offers invaluable insights into technological adoption, online community engagement, and gaming psychology, crucial for understanding contemporary digital trends. The interplay between advanced technology and Esports provides a unique perspective on the diffusion of innovation in the digital space.

## 3 Materials and methods

### 3.1 Procedure

The foundational principles of SNA, detailed by Wasserman and Faust (1994), include the interdependence of entities and their relational matrix, the role of ties as conduits for resource exchange, the network’s influence as a determinant of individual behaviors, and the persistence of these relational patterns over time. This paper utilizes ego network analysis, which centers on the network from the viewpoint of an individual entity, the ‘ego,’ encompassing the ‘ego,’ their direct connections—'alters,’—and the ties among these alters, forming what is also termed neighborhood networks (Hayat and Mo, 2015).

For our study, we gathered data from a subset of geocoded Twitter user pairs linked by ‘follow’ relationships, focusing on 50,000 users connected to the @IGN handle—a hub in the gaming news arena, indicative of a gaming-interested populace. We cataloged the locations and UI languages of these users, employing a bespoke scraper to mine data via Twitter’s streaming API, as per Rossi and Giglietto (2016). This API provides real-time access to tweets and associated metadata. Given the potential for data collection disruptions, we acknowledge, following Bruns and Stieglitz (2013), a margin of error, treating our dataset as an approximate rather than absolute representation of the stated data.

### 3.2 Measures

Geo-location data was harvested from our dataset, capturing the geographical coordinates of each participant and their connections, contingent on their use of GPS-enabled devices. These coordinates were translated into specific countries using the Google Maps Geocoding API, thus locating each participant and their network. Additionally, the API facilitated the measurement of distances in kilometers between participants and their connections.

We computed the Effective Network Size (ENS) for each participant to evaluate the scope of their Twitter-based networks. ENS quantifies “the number of direct contacts (alters) a focal participant (ego) has, adjusted by the alters’ connections among themselves, reflecting the concept of non-redundancy in social ties” (Burt, 1992 as cited in Lesser et al., 2017, p. 1612). Despite debates on the efficacy of weak ties versus non-redundancy, our methodology adheres to the measures validated by Burt (1992) and Anderson (2008).

The calculation of ENS, which serves as the independent variable in our analysis, was conducted through the UCInet software (Borgatti et al., 2002). ENS is calculated as follows: “Ego *v_i_*’s ENS in *G^S^* is denoted by the equation below, where *j* indexes all of those with whom ego *v_i_* has contact; and *q* is every third *v_q_* individual (other than *v_i_* or *v_j_*). The quantity (*p_iq_m_jq_*) inside the brackets is the level of redundancy between a given ego and a particular alter *v_j_* (Burt, 2009 from Lesser et al., 2017, p. 9).


$$\mathrm{effective}:\mathrm{size}\left(v_{i}\right)=\underset{j}{\sum }\left[1-\underset{q}{\sum }\rho_{iq}m_{jq}\right]$$

In order to calculate the ENS, we have collected the following data for each of our participants: (1) a list of his/her followings (2) a list of the followings of each of the twitter users our participants follow.

Creativity score was defined as the propensity of a participant to utilize a specific hashtag in its emerging stages, prior to its peak popularity as evidenced by Google Trends As discussed above, we focused the topic of Esports, derived from ‘electronic sports’, denotes the structured and competitive realm of video gaming, often involving professional participants and teams adhering to established rules and protocols. These contests span from regional skirmishes to grand international tournaments and are disseminated to vast global audiences via diverse digital mediums. With its burgeoning popularity, Esports not only parallels the ascent of video game culture and technological evolution but also redefines conventional paradigms of sportsmanship, athleticism, and socio-cultural engagement. Specifically, we looked at Google’s 2023 year in search, and examined the top 5 games ranked in this report,^1^ which are: Hogwarts Legacy; The Last of Us; Connections; Battlegrounds Mobile India; and Starfield. The creativity score was calculated based on the distance of when the user tweeted a tweet using one of these terms, and the peak day for that term. Higher score means that that the user tweeted further ahead from when the term reached its virality. This early adoption and utilization are indicative of a user’s ability to foresee, setting them apart from the majority who engage with content once it has gained broader recognition.

## 4 Results

ENS score, and Average Distance were are correlated with creativity score (see Table 1). To investigate how Average Distance moderates the association between ENS score and creativity an OLS regression is estimated (see Table 2), predicting the creativity score. The main independent variables are ENS score, and Average Distance (between the ego and it alters). The model proved to be statistically significant, accounting for 13% of the variation in the creativity scores. Both ENS score (*partial r* = 0.48, *p* < 0.001), and Average Distance (*partial r* = 0.36, *p* < 0.001) are positive predictors of the creativity score, thus affirming H1 (see Table 2).

**Table 1:** The association between ENS, creativity, and Average Distance.

	Creativity	ENS	Average Distance
Creativity	-	0.46***	0.36***
ENS	-	-	0.09***
Average Distance	-	-	-

Pearson’s R coefficients. *p < 0.05, **p < 01, ^***^p < 0.001.

**Table 2:** The interaction between Average Distance and ENS.

	(1) Main effects		(2) Interaction	
	*B* (SE)	*β*	*B* (SE)	*β*
ENS	0.48 (0.01)***	0.16	0.46 (0.01)***	0.13
Average Distance	0.36 (0.01)***	0.12	0.32 (0.01)***	0.09
ENS X Average Distance			0.24 (0.03)***	0.05
Constant	2.87 (0.001)***		2.43 (0.001)***	
*N*	50,000		50,000	
*R* ^2^	0.13		0.15	

^***^p < 0.001.

The analysis further showed (see Table 2) a notable interaction effect between the Effective Network Size (ENS), and Average Distance (*partial r* = 0.24, *p* < 0.001), indicating that the impact of ENS score on creativity is contingent upon the Average Distance (between the ego and it alters); thus, affirming H2.

**Figure 1:**
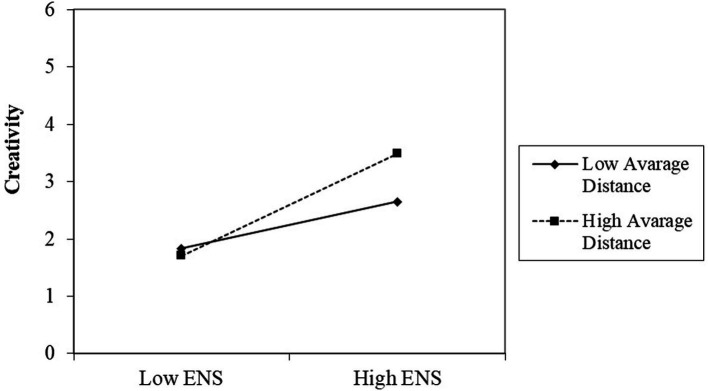
Interaction effect between ENS and average distance.

Simple slopes were tested for the interplay between ENS and creativity, for participants with high average distance, vs. participants with low average distance. For participants with high average distance a stronger correlation between ENS and creativity (b = 0.24, SEb = 0.03 *β* = 0.05, *p* < 0.001) was found, when compared to participants with low average distance. This interaction is depicted in Figure 1. The plotting of this data was conducted following the procedure proposed by Aiken and West (1991), to plot the interaction effects. Specifically, the plot visualize the distinction between individuals with low average distance, and those with average distance (the cutoff point for dividing individuals into this groups was the median distance score).

## 5 Discussion

Prior research has linked social capital and creative output with one’s network of social ties, yet there appears to be a gap in the literature concerning the specific impact of online social networks on creativity. Leveraging Twitter, this study explores social and psychological dynamics previously elusive to academic inquiry.

This investigation offers an effort to rigorously assess the relationship between individuals’ Effective Network Size (ENS) on Twitter and their creativity. Our findings align with Burt’s (2004) assertions, suggesting a positive correlation between ENS and creativity, challenging earlier conclusions by Zhou et al. (2009) and Perry-Smith (2006), who found no such link within non-digital contexts. This divergence highlights the distinctive nature of online versus offline network interactions.

By demonstrating a link between digital networking and creative activity, our research enriches the domain of online social network studies. Moreover, it advances the methodological landscape, employing direct data extraction from Twitter, thus avoiding the potential biases inherent in self-reported data (Parry et al., 2021).

Furthermore, the findings indicate that average distance (between the ego and its alters) moderates the correlation between ENS and creativity. When average distance was higher, ENS was more strongly correlated with creativity. Diverse social connections, as illuminated by sociologists like Burt (1992) and Granovetter (1977), offer individuals access to a broad spectrum of information and perspectives. Burt’s theory of structural holes suggests that those bridging gaps between different social groups act as brokers, combining disparate pieces of information in novel ways, thereby fostering creativity. Granovetter’s work on the strength of weak ties posits that distant connections or acquaintances often provide more novel information than close ties, as the latter frequently share similar knowledge and perspectives. This exposure to varied ways of thinking, especially when interacting with people from different backgrounds, enhances cognitive diversity, challenging existing beliefs and stimulating innovative thought.

Furthermore, the geographical distribution of one’s network (as evident by higher average distance) amplifies these benefits. Different regions come with their unique cultures, challenges, and ways of thinking. Engaging with geographically remote connections allows individuals to gain insights rooted in diverse local contexts, offering a richer array of ideas. This geographical diversity, combined with the cognitive diversity from varied social connections, reduces the risk of echo chambers and promotes the combination and recombination of ideas, which is often at the heart of innovation.

While no study is without limitations, several constraints within the study warrant recognition. Primarily, the research identified a correlational, rather than causative, link between ENS and creative output. Prospective investigations could rectify this by evaluating changes in creative levels pre- and post-modifications to users’ Twitter networks. Additionally, by leveraging social media platforms’ recommendation algorithms to introduce users to contacts outside their existing network, future research could explore the resultant variations in their creative capacities.

The second limitation of the study is that it examined Twitter only. Future studies should examine the findings on other online social networking sites and on different demographics. For instance, it would be interesting to examine the relationship between ENS and creativity on other age groups. One possibility could be children who use TikTok, a popular social media app for making and sharing short videos (Zhang et al., 2019). In addition, researchers could study the Chinese Twitter-like Weibo (Zhang and Pentina, 2012), and thereby explore any cultural gaps (Choi et al., 2011).

Thirdly, this study has primarily focused on analyzing creativity through the lens of utilizing new information in a meaningful way, before this information becomes widely recognized or adopted by others. While this approach offers valuable insights (Runco, 2007), it limits the scope of our understanding of creativity. Creativity manifests in diverse forms and through various processes that are not solely reliant on the novelty of information. Future studies should aim to broaden the examining of relationship between ENS and creativity, by examining other dimensions of creativity. Fos instance, after assessing the ENS of individuals (through Twitter or other platforms), individuals can be asked to complete a survey which assess acceptable scales of creativity, such as the Torrance Tests of Creative Thinking (Torrance, 1966); or the Creative Achievement Questionnaire, developed by Carson et al. (2005); which has been employed in numerous studies to gauge creative success. These measures can provide a more holistic understanding of creativity, encompassing its multifaceted nature and its manifestation in different contexts and fields.

Lastly, the dynamic interplay between social media algorithms and the study’s findings on Effective Network Size (ENS) and creativity, especially within Esports, underscores the algorithms’ role in amplifying the visibility and engagement of creative content. Algorithms prioritize content based on user engagement and network connections, which could magnify the observed positive correlation between ENS and creativity by ensuring wider reach for individuals with larger, non-redundant networks. However, this relationship also highlights concerns about the generalizability of results across different platforms with distinct algorithms and over time, as algorithmic changes could alter the effectiveness of leveraging online networks for creativity. This evolving digital landscape suggests the need for ongoing research to understand how social media’s algorithmic dynamics influence the creative potential of non-redundant social ties, offering insights for maximizing creativity in the face of changing online environments. Future studies could address this challenge through the study of creativity and ENS by unobtrusive methods; for example, using a Semantic Network Model to measure users’ creativity on social networking sites (e.g., Yu et al., 2016).

## 6 Conclusion

The current study offers an initial insight into how the makeup of online social networks can influence the advantages that non-redundant social connections offer to creative endeavors. These findings not only underscore the importance of online social fabric in fostering innovation in fields like Esports, but also hint at the broader applicability of social networking sites as platforms for enhancing creativity. Furthermore, the implications of using online social networking sites extend beyond the realm of social capital; they also have a significant impact on creative output. Thus, this research illuminates a novel perspective on the advantages that may arise from engaging with social networking platforms. It invites a reevaluation of these digital interactions, suggesting that they can serve as a catalyst for creativity, not just a means of communication or networking. This opens up new avenues for understanding how virtual connections fuel innovation and how strategic network expansion can foster creative development; hence offering valuable insights for leveraging online networks in cultivating creativity across various domains.

## Data availability statement

The raw data supporting the conclusions of this article will be made available by the authors, without undue reservation.

## Ethics statement

The study was approved by the Institutional Review Board of Reichman University, Herzliya. Written informed consent from the patients/participants or patients/participants’ legal guardian/next of kin was not required to participate in this study in accordance with the national legislation and the institutional requirements.

## Author contributions

TH: Writing – original draft.

## Footnotes

^1^ https://trends.google.com/trends/yis/2023/GLOBAL/?hl=en-US
